# Health spillover studies of long-term care insurance in China: evidence from spousal caregivers from disabled families

**DOI:** 10.1186/s12939-023-02001-6

**Published:** 2023-09-18

**Authors:** Wenjing Jiang, Hongyan Yang

**Affiliations:** 1 Center for Social Security Studies, Wuhan University, Wuhan, 430072 China; 2 School of Political Science & Public Administration, Wuhan University, Wuhan, 430072 China

**Keywords:** Long-term care insurance, Differences-in-differences, Disabled persons, Spousal care, Health

## Abstract

**Background:**

To alleviate the shortage of caregivers associated with disabled persons, China has implemented a pilot policy for long-term care insurance. This policy has the characteristics of "familialization" and "de-familialization" policy orientation, and it is indeed essential to clarify whether the policy has a positive spillover effect on the health of family caregivers, which is of great value to the pilot from local practice to national institutional arrangement.

**Methods:**

Based on the China Health and Retirement Longitudinal Study microdata and time-varying DID method, our study used the implementation of the pilot policy as a "quasi-natural experiment" to assess the health spillover effects of the pilot policy on family spousal caregivers.

**Results:**

This policy significantly improved the health of spousal caregivers, increasing self-rated health and life satisfaction, and reducing depression; Compared with female, urban and central-western spousal caregivers, male, rural and eastern spousal caregivers were "beneficiaries" in more dimensional health.

**Conclusions:**

Our research indicated that spousal caregivers of disabled people, particularly male, rural and eastern spousal caregivers, experienced positive health spillovers after implementing long-term care insurance. These results suggest that the imbalance between supply and demand of nursing staff could be solved in terms of de-familialization and familialization, spousal caregivers should be promoted to equally enjoy the policy benefits on gender, urban–rural and regions.

## Introduction

China's aging is accompanied by serious disability, and the once demographic dividend has been transformed into a thorny caregiver gap. According to statistics from China's Ministry of Civil Affairs and other departments, by the end of 2021, there were 267 million elderly people aged 60 and above in China, and about 44 million disabled and semi-disabled elderly people were facing the shortage of caregivers. Internationally, the care situation is equally dire, on average, 1.5% of Gross Domestic Product (GDP) was spent on all long-term care (LTC) services in 2018 in the Organisation for Economic Co-operation and Development (OECD), this equates to around USD 760 per capita [1], and in more than half of OECD countries, population ageing has been outpacing the growth of LTC supply [2]. The shortage of caregivers in China may be related to the following reasons: Declining family size, increasing geographical mobility and rising participation rates of women in labour market mean that there is a risk that fewer people will be willing and able to provide informal care in the future [2]. While, formal caregivers have less access to training, do not always have benefits such as paid annual leave, suffer from low job security, have less access to social protection, and pay is so low that temporary employment is common. In general, lacking continuity in formal caregivers further exacerbated the shortage of caregivers [2]. In this context, family caregivers, especially spouses, not only carry a heavy burden of caregiving, but also combine ageing, empty nesting and work-family balance, which are not conducive to their health sustainability and health equity.

To alleviate the shortage of caregivers associated with disabled persons, China officially introduced a pilot policy in 2016, requiring long-term care insurance (LTCI) pilots in 15 cities and 2 provinces. Espin-Anderson once specially introduced the concepts of "familialism" and "de-familialization" to characterize the state's attitude towards family. In general, "familialism" refers to the values and practical principles in which family is the primary responsible person in providing welfare for its members [3]. "De-familialization" refers to the provision of benefits through state or market in an attempt to reduce the burden of family care and reduce the welfare dependence of individuals on kinship. "Familialization" is relative to "de-familialization", which refers to strengthening family care function through state or market welfare interventions (such as time rights, allowances, etc.), although the family is still regarded as the primary responsibility for welfare, but the family itself has become the object of social policy support. In our opinion, the pilot generally presents a policy orientation of "familialization" and "de-familialization", This is reflected in two aspects: state provides nursing service payments (including hospitals and institutions) of about 70% for disabled persons who have reached severe disability, reducing the burden of family care, which reflects the policy orientation of "de-familialization"; Some pilot cities also support home-based kinship care services, using nursing allowances to encourage relatives to take the initiative to take care responsibilities, strengthening the family care function, which reflects the "familialization" policy orientation. According to data from the National Health Security Administration, as of 2023, more than 1.8 million disabled people have effectively enjoyed long-term care insurance treatment.

There is no consensus on whether long-term care insurance policy for has health spillovers on spousal caregivers in disabled groups. Some studies have reported that long-term care insurance policy has improved health status of family caregivers [4–8]. Other studies have also reported that long-term care insurance policy has not improved health of family caregivers [6, 8–11]. Based on Esping-Anderson's "familialism" policy system, Leitner subdivides "familialism" into four types [12]. The first type is “explicit familialism”, which strengthens family's care function through policies, such as time rights and nursing allowances, but lacks effective intervention of other subjects (state, market, society, etc.) and alternatives to family care; The second type is "free familialism", which not only provides relevant direct services, but also strengthens family care function through policies such as time rights and care allowances, giving families full choice; The third type is "implicit familialism", which has neither policies to strengthen family care function, nor related direct services to reduce the burden of family care. The family has a greater responsibility for welfare and is the ultimate supporter. The fourth type is "de-familialization", which refers to fact that the state and market reduce the family care burden and the welfare dependence of individuals on kinship relationships by providing direct services, so that the "familialization" structure is weak. As a central actor in the "familialization" structure, state can strengthen the family care function in the field of care through three types of policies: time rights, direct/indirect transfer of care benefits, and additional social rights. As a decentralized actor of "de-familialization" structure, the higher state intervention degree, the smaller family caregiving responsibility, and more pronounced burden reduction effect on family caregivers.

China's long-term care insurance policy objectives involve not only protecting the long-term care needs of disabled groups at the individual level, but also reducing the burden of family care, improving family development capacity and improving the welfare level of family members at the family level. According to Leitner's idea that care policy system should consider both the "familialization" structure and the "de-familialization" structure, impact of China's long-term care insurance policy on health of spousal caregivers should be positive in theory. This impact is embodied in two aspects: the "de-familialization" structure and "familialization" structure. On the one hand, long-term care insurance policy strengthens the "de-familialization" structure by providing institutional home care, so that family care is partially replaced by social care, employment potential of family caregivers is released, and the burden of family care affairs is reduced, thereby improving health status of spousal caregivers. On the other hand, long-term care insurance policy strengthens the familialization structure of disabled care field by providing kinship care subsidies, so that economic burden of family care is reduced, the original social consensus–care is a private domain problem –is adjusted, and the sense of social value of spousal caregivers is enhanced, thereby improving health status of spousal caregivers.

Taken together, our research hypothesis is China's long-term care insurance policy will have a positive welfare spillover effect on health of home caregivers of disabled people. Then, in practice, can long-term care insurance policy reflect health spillover effects on family caregivers (In this article, health spillover effects mean that long-term care insurance policy affect not only health of disabled people, but also health of family members, especially spouses)? Does this impact vary by gender division, urban- rural segregation and regional differences?

Therefore, based on the China Health and Retirement Longitudinal Study (CHARLS) microdata and time-varying DID method, our study took the implementation of long-term care insurance pilot policy as a "quasi-natural experiment”, evaluated health spillover effects of long-term care insurance pilot policy on spousal caregivers, and analyzed the heterogeneity of health effect on gender, rural–urban and regional differences.

## Materials and methods

### Data sources

Microdata is from the China Health and Retirement Longitudinal Study (CHARLS) data from 2011, 2013, 2015 and 2018. CHARLS collects a high quality nationally representative sample of Chinese residents ages 45, adopting multi-stage stratified PPS sampling. The baseline national wave of CHARLS is being fielded in 2011and includes about 10,000 households and 17,500 individuals in 150 counties/districts and 450 villages/resident committees. Individuals will be followed up every two years. All data will be made public one year after the end of data collection. CHARLS is similar to the Health and Retirement Study (HRS), its questionnaire includes the following modules: demographics, family structure/transfer, health status and functioning, biomarkers, health care and insurance, work, retirement and pension, income and consumption, assets (individual and household), and community level information.

**Fig. 1:**
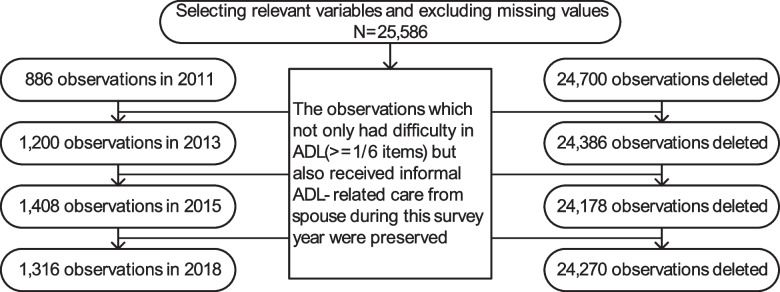
Sample selection process

In sample screening (See Fig. 1), after selecting variables relevant to our study and excluding missing values, 25,586 observations were preserved. And then, we identified disabled people who had difficulties in at least one of six items of activities of daily living (ADL), and the non-disabled people sample will be deleted. Finally, if disabled people reports that they received informal ADL-related care from their spouse during the survey, then their spouse will be identified as a spousal caregiver, and the non-spousal caregivers sample will be deleted. The final sample consisted of 886 spousal caregivers in 2011 wave, 1,200 in 2013 wave, 1,408 in 2015 wave, and 1,316 in 2018 wave. Because of the small number of pilot cities, short pilot time and database limitations, the sample size of our experimental group study is not large. We believe that small-sample research is also meaningful, there are many cases where large samples are not available and small samples are used to conduct research, and then solve reality problems. In previous literature using DID method to study the policy effects of pilot, proportion of samples in the experimental group was not high [13, 14].

Long-term care insurance data was taken from policy documents in pilot city. Based on these data, we were able to clarify pilot cities list and judge whether city carried out this policy change by the date of survey. The number of the first batch pilot cities is 15, and 53% of them carried out policy change after 2017. Table 1 reports on the evolution of pilot cities. We then matched city-level pilot data with individual-level data based on city and year variables.

**Table 1:** The first batch of long-term care insurance pilot cities and pilot time

Pilot cities	Implementation time	Pilot cities	Implementation time
Anqing	2017–03	Ningbo	2017–12
Changchun	2015–12	Qingdao	2012–7
Chengde	2017–12	Qiqihar	2017–10
Chengdu	2017–7	Shanghai	2017–1
Chongqing	2018–1	Shangrao	2016–11
Guangzhou	2017–8	Shihezi	2017–1
Jingmen	2016–11	Suzhou	2016
Nantong	2016–1	–	–

### Measures

#### Dependent variable: health

The dependent variable is health, charactering by self-reported health, depression and life satisfaction. In CHARLS, both self-reported health and life satisfaction range from 1 to 5, a higher value meaning better self-rated health and better life satisfaction. The score of depression ranges from 0 to 30, and we further assigned the score 0–9 to 1, 10–19 to 2, and 20–30 to 3, a higher value meaning worse depression. The multidimensional measures of health are based on previous studies [15–17].

#### Independent variable: LTCI

The independent variable is the implementation of long-term care insurance. The results of long-term care insurance range from 0 to 1, value 1 indicating that this city not only belongs to pilot list but also implemented the policy in survey year, otherwise, the value is 0.

#### Control variables

All analyses included a series of control variables associated with health of caregivers according to previous studies [15, 17, 18]. The sociodemographic characteristics include age, gender, education level and chronic disease; Work-life characteristics include employment status, retirement, alcohol consumption and smoking. Family characteristics include whether they live with their children, whether they have weekly face-to-face contact with their children, number of children and consumption per family. Social support characteristics include social pension insurance, social medical insurance and social activities. The variables analyzing heterogeneity were based on spousal caregivers’ gender differences, urban–rural differences and regional differences.

Table 2 reports descriptive statistical of main variables. Most spousal caregivers of disabled persons considered their health were between fair and good for mean was 2.843, depression was between no depression and mild depression for mean was 1.689, life satisfaction was between somewhat satisfied and very satisfied for mean was 3.116. Their average age was 64.08 and 52% were male. Their average education level was about sishu and 87.3% had chronic diseases. 60.6% currently were working and 37.1% were retired. Average daily drinking range was less than once. 48.4% had smoke ever and 44.3% were live with any child. 74.5% meet children weekly. Average number of children was 3.081. Logarithmic per capita household consumption was 8.889 per year. Among spousal caregivers, 44.2% currently were receiving public pension. 94.4% were covering by public health insurance. 40.7% participated in social activities. 81.4% had rural hukou. Average age of disabled persons was 64.39. And 95.1% disabled persons had chronic diseases.

**Table 2:** Definition and descriptive statistics

**Variables**	**Definition**	Mean	SD	Min	Max
Self-reported health	Very bad = 1, Fair = 2, Good = 3, Very good = 4, Excellent = 5	2.843	0.954	1	5
Depression	No depression = 1, Mild depression = 2, Severe depression = 3	1.689	0.754	1	3
Life satisfaction	Not at all satisfied = 1, Not very satisfied = 2, Somewhat satisfied = 3, Very satisfied = 4, Completely satisfied = 5	3.116	0.831	1	5
Treat*Post	Belong to the pilot city and the policy was implemented in the survey year = 1, Otherwise = 0	0.0225	0.148	0	1
Age	Age of spousal caregiver in survey year	64.08	9.506	22	95
Gender	Male = 1, Female = 0	0.520	0.500	0	1
Education	No formal education illiterate = 1, Did not finish primary school but capa = 2, Sishu = 3, Elementary school = 4, Middle school = 5, High school = 6, Vocational school = 7, Two/Three Year College/Associate degree = 8, Four Year College/Bachelor's degree = 9, Post-graduated (Master/ PhD) = 10	3.036	1.819	1	9
Chronic diseases	Yes = 1, No = 0	0.873	0.334	0	1
Currently working	Yes = 1, No = 0	0.606	0.489	0	1
Whether retired	Yes = 1, No = 0	0.371	0.483	0	1
Daily drinking range	None = 0, Less than once per day = 1, Once per day = 2, Twice per day = 3, More than twice per day = 4	0.583	0.967	0	4
Smoke ever	Yes = 1, No = 0	0.484	0.500	0	1
Live with any child	Yes = 1, No = 0	0.443	0.497	0	1
Meeting children weekly	Yes = 1, No = 0	0.745	0.436	0	1
Number of children	Number of living children	3.081	1.505	0	10
Household per capita consumption	Logarithmic per capita household consumption	8.889	0.974	4.357	13.24
Receiving public pension	Currently receiving = 1, Not receiving = 0	0.442	0.497	0	1
Covering by public health insurance	Yes = 1, No = 0	0.944	0.230	0	1
Participate in social activities	Yes = 1, No = 0	0.407	0.491	0	1
Urban–rural hukou	Rural hukou = 1, Urban hukou = 0	0.814	0.389	0	1
Age of disabled persons	Age in survey year	64.39	9.961	16	93
Chronic disease conditions in disabled persons	Yes = 1, No = 0	0.951	0.216	0	1

### Statistical analysis

Difference-in-difference (DID) method is a quasi-experimental technique that measures the causal effect of some nonrandom intervention [19]. In model construction process, we took pilot cities of long-term care insurance as experimental group, and other cities as control group. The year of policy implementation and beyond is used as experimental period, and remaining years are used as comparison expectation. Due to the different time of entering experimental period in each experimental group, we established time-varying DID method with reference to practices of other scholars [20]:

1 $${HEA}_{ict}={\alpha }_{1}+{\theta }_{1}{{Treat}_{ic}*Post}_{ct}+{\lambda }_{1}{Z}_{ict}+{\eta }_{c}+{\mu }_{t}+{\varepsilon }_{ict}$$

where ${HEA}_{ict}$ denotes the health status of spousal caregiver *i* who lives in city *c* in time *t*. ${Treat}_{ic}$ represents the pilot status of spousal caregiver *i* who lives in city *c*. ${Post}_{ct}$ represents the policy post status of city *c* in time *t*. ${\theta }_{1}$ measures the impact of this policy change on health. ${\lambda }_{1}$ is a vector of control variables ${Z}_{ict}$. ${\eta }_{c}$ and ${\mu }_{t}$ represent household and year fixed effects. ${\varepsilon }_{ict}$ represents random perturbations that affect health. Finally, the standard errors are clustered at city level to correct for possible autocorrelation and heteroscedasticity.

The parallel trend hypothesis is a key prerequisite for constructing time-varying DID method, which requires that the health trends of spousal caregivers in pilot cities and non-pilot cities must be parallel before policy implementation. Therefore, we used the event research method proposed by other scholars [21] to establish a parallel trend test model:

2 $${HEA}_{ict}={\alpha }_{1}+{\theta }_{t}\sum_{-2}^{3}{{Treat}_{ic}*Post}_{ct}+{\lambda }_{1}{Z}_{ict}+{\eta }_{c}+{\mu }_{t}+{\varepsilon }_{ict}$$

where ${\theta }_{t}$ reflects the health disparities in pilot and non-pilot cities in the t year of the policy post. 3 years before policy and 3 years after policy, the data is very little, so we aggregated the data 3 years before into year -3, the data 3 years after into year 3 and consider year -3 as the base year. Other variables are synonymous with Eq. (1).

## Results

### Main results

Table 3 reports regression results obtained using time-varying DID model. The coefficient of Treat*Post was 0.3817 (significant levels is 1%) in self-reported health, -0.2605 (significant levels is 5%) in depression and 0.1827 (not significant) in life satisfaction, indicating this pilot policy significantly increased self-reported health, lowered depression, and raised life satisfaction, but effect in life satisfaction was not significant.

**Table 3:** The results of time-varying DID model

**Variables**	**Self-reported health**	**Depression**	**Life satisfaction**
Treat*Post	0.3817^***^	-0.2605^**^	0.1827
	(0.1312)	(0.1133)	(0.1270)
Live with any child	-0.0658	-0.0313	-0.0484
	(0.0882)	(0.0554)	(0.0700)
Meeting children weekly	0.0426	0.0053	-0.0639
	(0.0986)	(0.0687)	(0.0741)
Number of children	-0.0380	-0.0731	-0.0428
	(0.0472)	(0.0480)	(0.0747)
Household per capita consumption	-0.0507	0.0224	0.0132
	(0.0337)	(0.0232)	(0.0387)
Age	0.0087	-0.0338^***^	0.0523^***^
	(0.0190)	(0.0117)	(0.0128)
Gender	-0.1862	-0.1263	-0.2454
	(0.1311)	(0.0991)	(0.1608)
Education	-0.0376	-0.0322	0.0076
	(0.0441)	(0.0278)	(0.0345)
Chronic diseases	-0.1932	-0.0142	0.2083
	(0.1347)	(0.1024)	(0.1300)
Currently working	0.0606	-0.3043	-0.0656
	(0.2488)	(0.1858)	(0.2501)
Whether retired	-0.1274	-0.0883	-0.2279
	(0.2582)	(0.1944)	(0.2526)
Daily drinking range	0.0351	-0.0196	0.0914^***^
	(0.0363)	(0.0207)	(0.0328)
Smoke ever	0.2887^***^	-0.0317	0.0969
	(0.1047)	(0.0792)	(0.1351)
Receiving public pension	0.0903	-0.0725	-0.0132
	(0.0554)	(0.0443)	(0.0546)
Covering by public health insurance	-0.0977	-0.0503	0.1360
	(0.1019)	(0.0992)	(0.1426)
Participate in social activities	-0.0594	0.0019	0.0737
	(0.0506)	(0.0396)	(0.0554)
Fixed household	Yes	Yes	Yes
Fixed year	Yes	Yes	Yes
_cons	3.0426^**^	4.4536^***^	-0.4542
	(1.2113)	(0.8004)	(1.0282)
*N*	1812	1849	1727
*R*^2^	0.639	0.640	0.593

Note: Values in parentheses are cluster robust standard errors, *, **, and *** represent significant levels of 10%, 5%, and 1%, respectively

### Parallel trend test and robustness test

Table 4 reports parallel trend test results at 95% confidence interval with considering year -3 as base year. The estimate values are rarely significant (except life satisfaction in year -2), in year -2 and year -1 while estimate values are almost significant in year 0 and subsequent. Therefore, the sample generally passed parallel trend test.

**Table 4:** Parallel trend test

Variables	Self-reported health	Depression	Life satisfaction
$Treat*Post$ year -2	0.585	-0.209	0.552^**^
	(0.449)	(0.135)	(0.221)
$Treat*Post$ year -1	0.786	0.128	-0.479
	(0.586)	(0.163)	(0.308)
$Treat*Post$ year 0	0.274***	-0.367***	0.261^***^
	(0.052)	(0.037)	(0.065)
$Treat*Post$ year 1	0.868**	-0.411**	0.391^*^
	(0.385)	(0.182)	(0.212)
$Treat*Post$ year 2	0.136	0.526***	0.324
	(0.135)	(0.105)	(0.419)
$Treat*Post$ year 3	0.267	-0.109	0.016
	(0.374)	(0.136)	(0.240)
Control variables	Yes	Yes	Yes
Fixed household	Yes	Yes	Yes
Fixed year	Yes	Yes	Yes
_cons	2.891**	4.052***	-0.472
	(1.118)	(0.861)	(1.043)
N	1827	1889	1727
R^2^	0.639	0.633	0.595

*, **, and *** represent significant levels of 10%, 5%, and 1%, respectively. The values in parentheses are city-level clustering robust standard errors

We tested the robustness from two aspects. Firstly, we limited the age of disabled persons to 45 and above. Secondly, we limited disabled persons to chronic disease groups. Table 5 reports results of robustness tests. The results were consistent with those in time-varying DID model. Therefore, our results were generally robust.

**Table 5:** Robustness test

Variables	Disabled persons aged 45 and above			Disabled people have chronic diseases		
	**Self-reported health**	**Depression**	**Life satisfaction**	**Self-reported health**	**Depression**	**Life satisfaction**
Treat*Post	0.3828^***^	-0.2596^**^	0.1808	0.3701^***^	-0.2456^**^	0.1555
	(0.1323)	(0.1135)	(0.1269)	(0.1296)	(0.1150)	(0.1305)
Control variables	Yes	Yes	Yes	Yes	Yes	Yes
Fixed household	Yes	Yes	Yes	Yes	Yes	Yes
Fixed year	Yes	Yes	Yes	Yes	Yes	Yes
_cons	2.2987^**^	4.9396^***^	-0.4129	3.2447^***^	4.1496^***^	-0.6954
	(1.1101)	(0.7639)	(1.0567)	(1.2149)	(0.7712)	(1.0072)
N	1797	1834	1714	1735	1770	1653
R^2^	0.639	0.644	0.592	0.643	0.642	0.597

Values in parentheses are cluster robust standard errors, ** and *** represent significant levels of 5% and 1%, respectively

### Analysis of heterogeneity

From our main results, Long-term care insurance policy has positive health spillover effects on spousal caregivers. Based on heterogeneity of health spillover effects, it is more conducive to improving long-term care insurance policy framework that match supply and demand adaptability. However, previous studies lack in-depth discussion on gender, urban–rural and region heterogeneity. Therefore, we explored health spillover effects on gender (Table 6), urban–rural (Table 7) and region (Table 8).

**Table 6:** Gender heterogeneity of spousal caregivers

Variables	Male			Female		
	**Self-reported health**	**Depression**	**Life satisfaction**	**Self-reported health**	**Depression**	**Life satisfaction**
Treat*Post	0.5121***	-0.1492	0.2883*	0.4452	-0.4127***	0.2837
	(0.1388)	(0.1801)	(0.1478)	(0.2747)	(0.1399)	(0.2677)
Control variables	Yes	Yes	Yes	Yes	Yes	Yes
Fixed household	Yes	Yes	Yes	Yes	Yes	Yes
Fixed year	Yes	Yes	Yes	Yes	Yes	Yes
_cons	6.6394	-3.6825	-10.8763	0.6244	4.5640	9.6500
	(14.7416)	(10.1005)	(16.1910)	(8.5841)	(6.6671)	(17.5413)
N	785	803	755	685	693	644
R^2^	0.711	0.670	0.631	0.663	0.675	0.662

Values in parentheses are cluster robust standard errors, * and *** represent significant levels of 10% and 1%, respectively

**Table 7:** Urban–rural heterogeneity of spousal caregivers

Variables	Urban			Rural		
	**Self-reported health**	**Depression**	**Life satisfaction**	**Self-reported health**	**Depression**	**Life satisfaction**
Treat*Post	0.3078	-0.5090***	0.4543	0.4525**	-0.1222	0.2197*
	(0.1942)	(0.1711)	(0.3070)	(0.1982)	(0.1414)	(0.1297)
Control variables	Yes	Yes	Yes	Yes	Yes	Yes
Fixed household	Yes	Yes	Yes	Yes	Yes	Yes
Fixed year	Yes	Yes	Yes	Yes	Yes	Yes
_cons	-9.3806	5.8685*	-3.7025*	4.8345***	4.0359***	0.8659
	(7.0172)	(3.3630)	(2.1832)	(1.6469)	(1.1455)	(1.4296)
N	272	280	266	1431	1459	1354
R^2^	0.707	0.712	0.667	0.634	0.629	0.590

Values in parentheses are cluster robust standard errors, *, **, and *** represent significant levels of 10%, 5%, and 1%, respectively

**Table 8:** Regional heterogeneity of spousal caregivers

Variables	Eastern region			Central and western regions		
	**Self-reported health**	**Depression**	**Life satisfaction**	**Self-reported health**	**Depression**	**Life satisfaction**
Treat*Post	0.6616***	-0.4542*	0.0285	0.4660	-0.0668	0.1594
	(0.1916)	(0.2230)	(0.1226)	(0.3206)	(0.2034)	(0.3111)
Control variables	Yes	Yes	Yes	Yes	Yes	Yes
Fixed household	Yes	Yes	Yes	Yes	Yes	Yes
Fixed year	Yes	Yes	Yes	Yes	Yes	Yes
_cons	14.4115***	-1.5281	1.9923	1.4876	5.1499***	-1.1934
	(2.9332)	(2.4322)	(1.7005)	(0.9067)	(0.7871)	(1.2114)
N	358	366	345	1326	1352	1259
R^2^	0.715	0.670	0.644	0.623	0.634	0.582

Values in parentheses are cluster robust standard errors, * and *** represent significant levels of 10% and 1%, respectively

Table 6 reports the Gender heterogeneity results. The pilot policy significantly increased self-reported health and life satisfaction for women and lowered the depression for men. Male spousal caregivers benefited more.

Table 7 reports the urban–rural heterogeneity results. The pilot policy significantly increased self-reported health and life satisfaction for rural residents and lowered the depression for urban residents. Rural spousal caregivers benefited more.

Table 8 reports the regional heterogeneity results. The pilot policy significantly increased self-reported health and lowered depression for eastern region and had no significant impact for central and western regions. Spousal caregivers in eastern region benefited more.

## Discussion

Our finding that long-term care insurance policy has positive health spillovers on spousal caregivers is consistent with previous studies. Taking Qingdao as an example, some studies have found that long-term care insurance policy significantly reduces depression tendency of middle-aged and elderly people (including caregivers), and has a positive effect on health [5]. Taking Taiwan as an example, long-term care insurance home care services have been found to be significantly associated with improving self-reported health of elderly family caregivers [6]. Cross-border studies have also found that respite care and nursing allowances reduce deterioration of self-reported health status of family caregivers [4]. In Spain, studies have found that access to home care allowances is significantly associated with higher levels of health-reported quality of life for family caregivers [7]. In Japan, studies have found that depression rate of Japanese family caregivers has decreased after the introduction of long-term care insurance policy [8].

The possible reasons why long-term care insurance policy has positive health spillovers on spousal caregivers come from two aspects. on the one hand, policy provides "de-familialization" institutional home care services for disabled persons replacing home care originally undertaken by spouse, which greatly reducing transactional care burden on spousal caregivers and releasing formal labor employment opportunities. This conclusion coincides with research of other scholars at home and abroad: Evidence based on Canadian found that publicly funded home care programs reduce informal home care [22]; Evidence based on South Korea found that beneficiaries of long-term care insurance policy were more likely to choose institutional care [23]; Based on Chinese evidence, it is found that long-term care insurance policy has increased proportion of disabled elderly families choosing social care mode, while proportion of family care mode has decreased [24]; Moreover, long-term care insurance significantly increases probability of non-agricultural and out-of-home employment and potential working hours of rural female caregivers [25].

On the other hand, policy encourages the development of family-based home kinship care services, directly provides nursing allowances for relatives of disabled persons, and reduces financial care burden of spouses through direct cash payments to spouses, while also recognizing social value of spousal care labor. Existing studies provide supporting evidence for this conclusion: researchers have found across countries that respite care and nursing care allowances reduce deterioration of family caregivers' health more than other public support policies for informal caregivers [4]. The study in Spain also found that access to home care allowances was significantly associated with a higher level of health-related quality of life for family caregivers, which favored the role of caregiver in optimal conditions [7].

We consider that the reason why male spousal caregivers benefited more from long-term care insurance policy stem from the traditional division of labor leading to women becoming "victims" of family care [26], unequal labor market opportunities contribute to the gender health gap [27]. It can be discussed here on a case-by-case basis. firstly, when families of disabled persons choose institutional home care (reflecting the de-familialization structure), family care is partially replaced by social care, and women are given the right to choose formal employment or family care. If spousal caregivers choose formal work, men have fewer difficulties in the labour market, while women tend to experience workplace discrimination, low job suitability and low income. If spousal caregivers choose to continue caring for the family (including home-based kinship care that reflects the familialization structure), men are more physically fit to tolerate an overload of transactional care activities. Therefore, male spousal caregivers benefited more from long-term care insurance policy.

In our opinion, the reason why rural spousal caregivers benefited more from long-term care insurance policy was mainly because the accessibility of long-term care services has improved in rural areas. The widespread unequal distribution of long-term care resources between urban and rural areas limits the accessibility of long-term care services in rural China, rural spousal caregivers’ health damage caused by care burden is severe. The accessibility of long-term care services in rural areas was improved after the implementation of policy, spousal caregivers can not only reduce the administrative burden through institutional home care services (reflecting de-familialization), but also reduce economic burden through home-based kinship care services (reflecting familialism), thereby producing positive health spillover effects.

As for why spousal caregivers in eastern region benefited more, we consider regional economic differences have produced conditional differences for de-familialization and familialization is the reason. The economic development level in China is characterized by a higher level in eastern region and a lower level in central and western regions. The eastern region has conditions not only for familialization but also for de-familialization, in other words, home-based kinship care and institutional home care can be achieved in eastern region. However, the central and western regions mainly have the conditions of familialization, many provinces do not have financial ability to provide institutional home care.

## Conclusions

Maintaining health attention to spouses of persons with disabilities, especially those who have assumed family disability care responsibilities, is critical to achieving health equity. This study examines health effects of long-term care insurance policy for disabled persons on their spousal caregivers, and concludes the following: (1) Overall, long-term care insurance policy has positive health spillovers to spousal caregivers, significantly improving health of spousal caregivers, increasing self-rated health and life satisfaction and reducing depression. This conclusion is still true after parallel trend test and robustness test. (2) The positive health spillovers to spousal caregivers is heterogeneous on gender, urban–rural and regional differences. Male, rural and eastern region spousal caregivers benefit more from long-term care insurance policies. Based on findings, our study provides the following policy implications. First, long-term care insurance policies should focus on solving the imbalance between supply and demand of nursing staff. In terms of de-familialization, the government-school-institution joint training model of nursing staff can be adopted, with government providing subsidies and policy loans, schools providing professional training and nursing institutions providing employment opportunities, so that surplus labor force in urban -rural areas and eastern and central-western regions can effectively expand the full-time nursing team. In terms of familialization, psychological intervention and respite services should be provided for family caregivers, to improve their mental stress, physical burden and multidimensional health. Secondly, long-term care insurance policy should pay attention to welfare stratification associated with health spillovers in gender, urban–rural and regions, and promote equal enjoyment of benefits on gender, urban–rural and regions.

## Data Availability

CHARLS data are available at https://charls.charlsdata.com/users/profile/index/en.html (requiring a simple application).

## References

[1] OECD. Spending on long-term care. 2020. https://www.oecd.org/health/health-systems/Spending-on-long-term-care-Brief-November-2020.pdf. Accessed 7 Aug 2023.

[2] OECD. Health at a Glance 2021. 2021. 10.1787/ae3016b9-en.

[3] Tan X. Familism in the Welfare Regimes: Concept, Connotation and Controversy. Ningxia Soc Sci. 2020;57–66. https://kns.cnki.net/KXReader/Detail?invoice=v94Z9HakW33GZu8oo2J9zxNQjLmMrlaIhrapfgt%2FiGsxpkt%2BkuoOvbTQPXNAZfpujTgokJaKhvdfsJJKREk2%2BCakQKhfEWYpXov8llBR8ur%2BZbyAKaXR860ONr%2B2eDAlgmHrZ4UQYHhYHqED5DHSeKmME4JkQu61ML50Izwmzoc%3D&DBCODE=CJFD&FileName=LXSK202006009&TABLEName=cjfdlast2020&nonce=F9A97853B5004D20A6CA849E36CE4A7E&TIMESTAMP=1688553301590&uid.

[4] Calvó-Perxas L, Vilalta-Franch J, Litwin H, Mira P, Garre-Olmo J (2021). A longitudinal study on public policy and the health of in-house caregivers in Europe. Health Policy.

[5] Ma C, Yu Q, Song Z, Chen H. Long-term care insurance, the control of medical expenses and “Value-Based Health Care". China Ind Econ. 2019;12:42–59. 10.19581/j.cnki.ciejournal.2019.12.003.

[6] Chen M-C, Kao C-W, Chiu Y-L, Lin T-Y, Tsai Y-T, Jian Y-TZ, Tzeng Y-M, Lin F-G, Hwang S-L, Li S-R (2017). Effects of home-based long-term care services on caregiver health according to age. Health Qual Life Outcomes.

[7] del Río Lozano M, García-Calvente MdM, Calle-Romero J, Machón-Sobrado M, Larrañaga-Padilla I. Health-related quality of life in Spanish informal caregivers: gender differences and support received. Qual Life Res. 2017;26:3227–38. 10.1007/s11136-017-1678-2. 10.1007/s11136-017-1678-2 28780713

[8] Oura A, Washio M, Arai Y, Ide S, Yamasaki R, Wada J, Kuwahara Y, Mori M (2007). Depression among caregivers of the frail elderly in Japan before and after the introduction of the Public Long-Term Care Insurance System. Z Gerontol Geriatr.

[9] Miyawaki A, Kobayashi Y, Noguchi H, Watanabe T, Takahashi H, Tamiya N (2020). Effect of reduced formal care availability on formal/informal care patterns and caregiver health: a quasi-experimental study using the Japanese long-term care insurance reform. BMC Geriatr.

[10] Schmitz H, Westphal M (2015). Short- and medium-term effects of informal care provision on female caregivers’ health. J Health Econ.

[11] Schmitz H, Stroka MA (2013). Health and the double burden of full-time work and informal care provision — Evidence from administrative data. Labour Econ.

[12] Leitner S (2005). Conservative familialism reconsidered: The Case of Belgium. Acta Politica.

[13] Lu C, Lu W, Wan W. Does long-term care insurance improve the subjective well-being of middleaged adults--a bidirectional analysis of positive and negative emotions. Soc Sec Stud. 2023;15–32. https://kns.cnki.net/kcms2/article/abstract?v=KTWJcyGkBkDRfFBg4aIPYChvTxZnRKy2W8pNgcH0kysA9wsEwXFOOKDBVkRRJfT_jPEhWPU9m6uxjsRO0ugJHllkIQ69A5lZmPs8x3u4tLHJwXbtBqyy82Dp7Eel6A2aFmLmvkWvGlA=&uniplatform=NZKPT.

[14] Zhenyu Z. The effect of the long-term care insurance on family care for the elderly. Chin J Popul Sci. 2023;37:97–114, :https://kns.cnki.net/kcms2/article/abstract?v=KTWJcyGkBkAe10Lzj0at4Ug24C2vLq1QPri3hhJyLfskbViV5kPRuI_7BBc4xRNxrYB6kaP_T6MDI3AmNAI6c1AyRalTGIzMqZo_WH8UJXztHtKgzaly-WAkI2ZVBLyU&uniplatform=NZKPT&flag=copy.

[15] Albert-Ballestar S, García-Altés A (2021). Measuring health inequalities: a systematic review of widely used indicators and topics. Int J Equity Health.

[16] Moreira I, Ferrer M, Vilagut G, Mortier P, Felez-Nobrega M, Domènech-Abella J, Haro J-M, Alonso J (2023). Social inequalities in mental and physical health derived from the COVID-19 pandemic in Spain beyond SARS-CoV-2 infection. Int J Equity Health.

[17] Meisters R, Putrik P, Westra D, Bosma H, Ruwaard D, Jansen M (2023). Two sides of the same coin? Absolute income and perceived income inadequacy as social determinants of health. Int J Equity Health.

[18] Kaikeaw S, Punpuing S, Chamchan C, Prasartkul P (2023). Socioeconomic inequalities in health outcomes among Thai older population in the era of Universal Health Coverage: trends and decomposition analysis. Int J Equity Health.

[19] Angrist JD, Krueger AB. Chapter 23 - Empirical Strategies in Labor Economics. In Handbook of Labor Economics. Ashenfelter OC, Card D, Eds. Vol. 3. Elsevier. 1999. p. 1277–1366.

[20] Beck T, Levine R, Levkov A (2010). Big bad banks? The winners and losers from bank deregulation in the United States. J Financ.

[21] Jacobson LS, LaLonde RJ, Sullivan DG. Earnings losses of displaced workers. The American Economic Review 1993; 83:685–709:http://www.jstor.org/stable/2117574.

[22] Stabile M, Laporte A, Coyte PC (2006). Household responses to public home care programs. J Health Econ.

[23] Kim H, Kwon S, Yoon N-H, Hyun K-R (2013). Utilization of long-term care services under the public long-term care insurance program in Korea: Implications of a subsidy policy. Health Policy.

[24] Cai W, Liu H, Shen X. Long-term care lnsurance, Residents' care choices and intergenerationa. Policy Evaluation Based on Pilot Cities in ChinaSuppor. Economic Perspectives 2021, 48–63, :https://kns.cnki.net/KXReader/Detail?invoice=vLhgtJYFaoB07O%2F%2BkTqGz5oxvBD3eGvV3c2FGYBIDUqkxYXDeEOEo9rnTvjKsAoVsanCqL7AemIcGIUKZ4n0jS7Dr1WGvSIFTj9bY%2Bwg3BKmFsealyNdhuloanbGixXS2SwtXAvmawb3N68qT%2BUS%2BKG0QJwQR%2BatK2DtyVdwq5E%3D&DBCODE=CJFD&FileName=JJXD202110004&TABLEName=cjfdlast2022&nonce=E994C0C8215C4178AE30752BCDEF281C&TIMESTAMP=1688383918492&uid=.

[25] Yu X, Huang J, Kang Z, Yu W. Elderly care security and female labor participation: policy effects evaluation of long-term care insurance in Rural China. Chinese Rural Economy 2021; 125–144:https://kns.cnki.net/KXReader/Detail?invoice=gHLVF%2BIhaHGaa2UK85o3t7uhvzpfSwKTKrQxxJuzkj1HuJabBGVeRpki2bq5e%2B3L1Bf%2F1CwinQGcUJ8J8Tdn1K7cBQ%2F92ePeZulbn5Ylm1ApRALNDV52WnB81ATULAYimaWb0AqTpm153KC32lAXXMKcjqSN%2FZODytNBURiePMs%3D&DBCODE=CJFD&FileName=ZNJJ202111008&TABLEName=cjfdlast2022&nonce=3620CB7A025D471E80801F2038BC4CC3&TIMESTAMP=1688385256129&uid=.

[26] Miller B, Cafasso L (1992). Gender differences in caregiving: fact or artifact?1. Gerontologist.

[27] Gueltzow M, Bijlsma MJ, van Lenthe FJ, Myrskylä M. The role of labor market inequalities in explaining the gender gap in depression risk among older US adults. Soc Sci Med. 2023;332:116100. 10.1016/j.socscimed.2023.116100. 10.1016/j.socscimed.2023.116100 37515952

